# Differing In Vitro Rooting and Flowering Responses of the Persian Violet to Low and High UV-C Irradiation

**DOI:** 10.3390/plants10122671

**Published:** 2021-12-04

**Authors:** Saowaros Phanomchai, Sompoch Noichinda, Yongsak Kachonpadungkitti, Kitti Bodhipadma

**Affiliations:** 1 Division of Agro-Industrial Technology, Faculty of Applied Science, King Mongkut’s University of Technology North Bangkok, Bangsue, Bangkok 10800, Thailand; ph.saowaros@gmail.com (S.P.); sompoch.n@sci.kmutnb.ac.th (S.N.); 2 Department of Biotechnology, Faculty of Science and Technology, Thammasat University, Khlong Luang, Pathum Thani 12121, Thailand; yongsakk@hotmail.com

**Keywords:** *Exacum affine*, in vitro mutation, plant tissue culture, root initiation

## Abstract

Persian violet flowers are considered esthetically attractive, leading to the high economic value of this plant. Plant breeding is fundamental to crop improvement, and the induction of mutation by tissue culture technology in combination with irradiation has been beneficially applied to generate plants with novel desirable characteristics. In this research, single or double rounds of UV-C irradiations were carried out on plant tissue cultures to initiate the in vitro rooting and mutation of Persian violets. It was found that single low-intensity UV-C exposure, when applied to Persian violet microshoots for 4 h, could induce the maximum number of roots and the highest root length without the use of a plant growth regulator. Overall, the single and double UV-C irradiation of Persian violet microshoots led to 44 different types of Persian violet flower mutations. Under single high-intensity UV-C irradiation for 6 h, up to nine petals were initiated, whereas single low-intensity UV-C irradiation did not influence the morphological variation of Persian violet flowers. Thus, Persian violet microshoots respond differently in terms of in vitro rooting and flowering depending on the UV-C intensity and exposure duration. These outcomes may be applied to micropropagation and in vitro plant breeding.

## 1. Introduction

Generally, plant micropropagation has four stages including establishing aseptic cultures, multiplication, root induction, and acclimatization [1]. During in vitro multiplication, a shoot is produced and successfully remultiplied for prolonged cultivation. However, without root organogenesis, complete plantlet formation is impossible. To obtain the entire plant in this axenic condition, rooting of regenerated shoots is an essential step that may or may not require the use of external phytohormones during development on a culture medium, depending on the plant genotype [2,3].

In organ formation, various methods have been employed for the induction of adventitious roots in vitro. Plant growth regulators, especially auxin, either alone or in combination, are the most desirable root inducers [4,5]. To date, many substances, such as activated charcoal and ginger rhizome extract, have been used either alone or in conjunction with auxin to enhance the root number and length of micropropagated shoot explants [6,7]. However, finding other methods for root initiation under sterile conditions is necessary for reducing the cost of chemicals used in rooting.

At present, the ornamental flower industry is expanding rapidly, leading to an increased demand for species diversity. Consumers are attracted to novelty, and the development of novel specific characteristics of flowers is one means of making them more attractive to customers. In this respect, plant tissue culture technology, which can be used for inducing variation in plants, is an essential tool for plant breeders. Induced mutation is a method that increases the rate of mutation above that of spontaneous mutation. Plant mutation induction based on tissue culture technology combined with physical mutagens (e.g., different types of radiation) is one of the effective methods for plant breeding. It offers a uniform mutagen, increases the mutation rate, allows the maintenance of disease-free plants, and only requires a short duration and a lower amount of space. Induced mutation by radiation thus increases the possibility of developing traits of economic importance in agriculture that are otherwise not found naturally or may have been lost during evolution [8,9].

UV-C is one of the physical mutagens that possibly causes genetic variation and enables the selection of traits as needed. Using UV-C in combination with plant tissue culture provides several benefits. In alfalfa callus, exposure to UV-C for 60 min increases drought tolerance [10]. Grape callus and moringa shoot cultured under sterile conditions showed elevated secondary metabolite accumulation and antioxidant activity, respectively, after UV-C irradiation [11,12]. Somaclonal variability was also observed in potato callus after UV-C radiation [13].

At present, to the best of our knowledge, there is no evidence in the literature confirming that UV-C can induce in vitro rooting of the Persian violet, and the effect of UV-C re-irradiation on the morphological variation of the Persian violet flower under sterile conditions has not been previously studied. Therefore, we aimed to investigate the effect of low- and high-intensity single or double (repeated) UV-C exposure on aseptic root induction and visible mutagenicity of flowers of the Persian violet in vitro.

## 2. Results

### 2.1. UV-C Induced Persian Violet Root In Vitro

**Figure 1:**
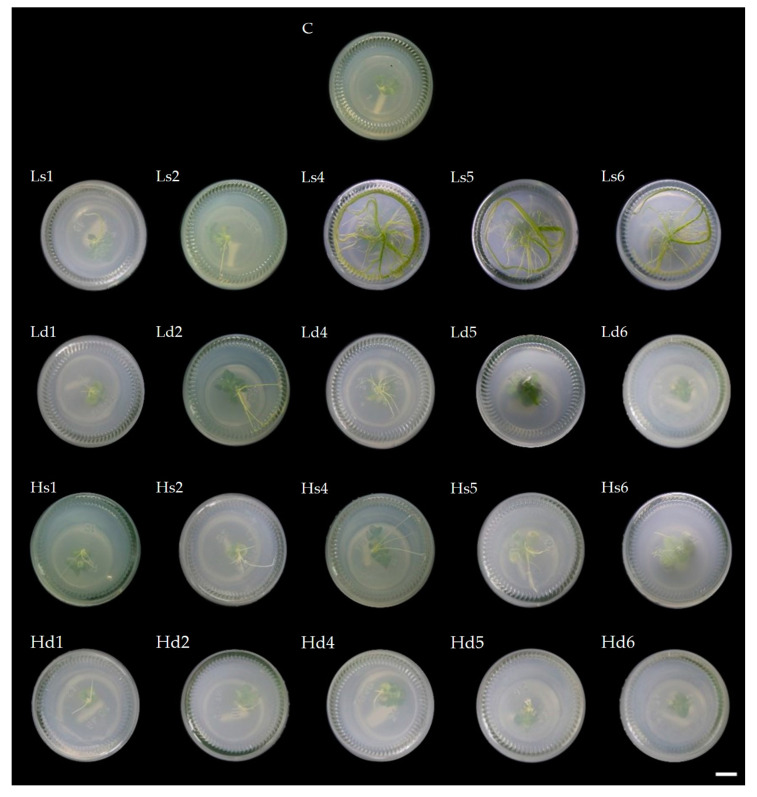
Effect of low- and high-intensity UV-C with single or double irradiation on Persian violet root initiation after subsequent culturing for 4 weeks: C = control; L = low-intensity; H = high-intensity; s = single irradiation; d = double irradiation; 1, 2, 4, 5, 6 = 1, 2, 4, 5, and 6 h (scale bar = 1 cm).

The Persian violet microshoots were treated in vitro with low- and high-intensity UV-C (30 and 234 µW/cm^2^, respectively) in single or double (repeated with a 24 h interval) rounds of irradiation for 0, 1, 2, 4, 5, or 6 h before culturing for 4 weeks on MS [14] basal medium. It was found that single or double exposure to low- and high-intensity UV-C affected Persian violet root induction under sterile conditions. A single low-intensity UV-C exposure for 4 h led to the most significant root number and root length (3.1 roots and 9.3 cm, respectively), as shown in Table 1 and Figure 1. It also gave the highest plantlet height (3.5 cm), and the number of shoots was up to 1.4 (Table 1); however, when treated with double high-intensity UV-C exposure, most plantlets had lower root numbers and a shorter length than those that had only a single round of low-intensity UV-C exposure.

### 2.2. UV-C Induced In Vitro Mutation of the Persian Violet Flower

**Figure 2:**
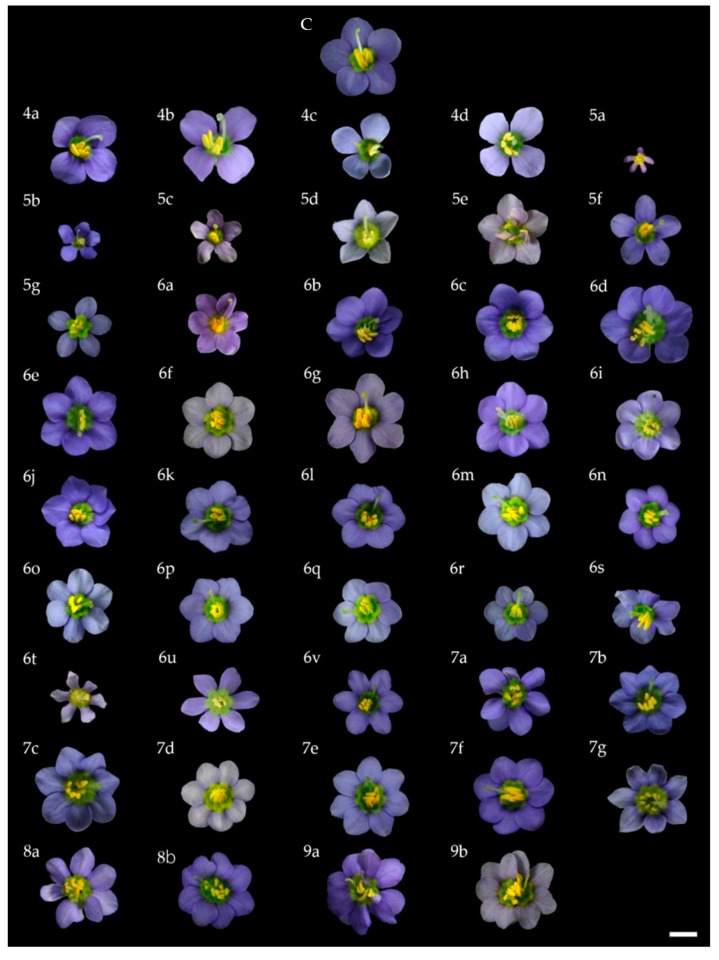
Effect of low- and high-intensity UV-C on Persian violet flower characteristics after irradiation and then culturing for 8 weeks: C = control; 4a–4d = 4 petals; 5a–5g = 5 petals; 6a–6v = 6 petals; 7a–7g = 7 petals; 8a–8b = 8 petals; 9a–9b = 9 petals (scale bar = 0.5 cm).

Following the in vitro treatment of Persian violet microshoots with low- and high-intensity UV-C in single or double rounds of irradiation for 0, 1, 2, 4, 5, or 6 h and subsequent culture, the microshoots showed good growth and started flowering around week 6. At the end of the experiment (week 8), a total of 948 flowers emerged from all treatments (Table S1). Interestingly, the highest percentage of in vitro Persian violet flowering was obtained (93.3%) in the group with a single exposure to high-intensity UV-C for 6 h (Table S2). The subsequent examination of the morphology revealed that 44 of the resulting flowers had morphological differences from ordinary unmutated flowers (Figure 2), representing 4.6% (Table S1), with the most significant percentage of flower mutation being 27.6% in the group with a single high-intensity UV-C irradiation for 5 h (Table S3). Furthermore, the group with a single exposure to high-intensity UV-C for 4 and 6 h had percentage mutation reductions of 21.2% and 19.6%, respectively (Table S3); however, a single exposure to low-intensity UV-C did not induce any morphological variation in Persian violet flowers (Table S3).

The resulting mutation affected the appearance of Persian violet flowers by changing their structure or form, for example, the flower diameter, number of petals, flower shape, petal color, and flower components compared to ordinary flowers (Figure 2, Table 2).

Persian violet flowers ordinarily have the following characteristics: diameter of around 0.8 ± 0.7 cm, 5 petals with violet-blue group color (93-B), five anthers with yellow-orange group color (14-A), and an emerging style approximately 4 mm long. After UV-C irradiation and culturing for 8 weeks, 44 Persian violet flowers were found to vary from the ordinary flowers, with 44 distinct patterns (Figure 2, Table 2).

**Figure 3:**
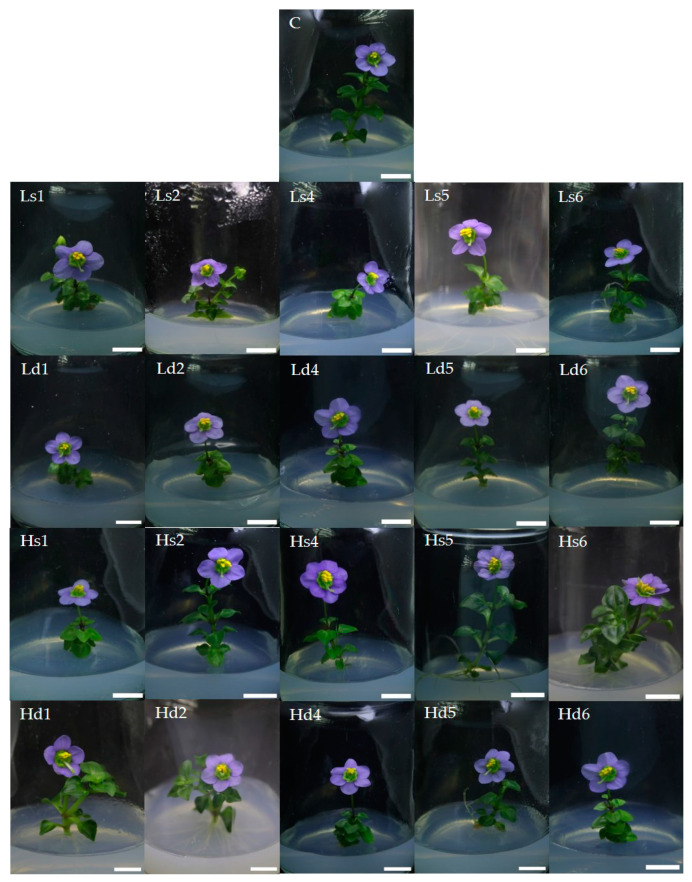
Effect of low- and high-intensity UV-C on Persian violet shoot height in vitro after subsequent culturing for 8 weeks: C = control; L = low-intensity; H = high-intensity; s = single irradiation; d = double irradiation; 1, 2, 4, 5, and 6 = 1, 2, 4, 5, and 6 h, respectively (scale bar = 1 cm).

A single 5 h exposure to high-intensity UV-C produced a maximum of 16 different flower mutations. In addition, the single 6-hour high-intensity UV-C exposure resulted in mutations with the highest number (9) of petals (Table 2). Nevertheless, when examining flower size, the number of flowers, and the number of petals (Table 3), it was found that a single high-intensity UV-C irradiation for 4–6 h, followed by culturing on MS basal medium for 8 weeks, resulted in statistically significantly higher values than other treatments but not for the highest height of in vitro Persian violet plantlets (Table 3 and Figure 3).

## 3. Discussion

### 3.1. Plantlet Establishment

Persian violet, an ornamental plant having small, purple flowers with yellow pollen in its center, is mainly grown as a houseplant [15]. For micropropagation, microshoot explants of the Persian violet (1 mm long) have previously been used for multiple shoot induction [16,17]. In the present research, 5 mm long microshoots were employed as explants to initiate the development of multiple shoots on MS basal medium as an alternative to adding BAP. Although this method provided a lower number of shoots, the new shoots were more prominent and vigorous, and the process was shorter in duration than that using BAP medium.

### 3.2. In Vitro Persian Violet Root Initiation

Rooting is an essential part of micropropagation that results in a whole plant after shootlet multiplication. In vitro root formation can occur either autonomously or by induction. While some explants do not require plant growth regulators for root regeneration, many depend on exogenous hormones to induce rooting [18,19]. The use of different radiation sources has recently made available new choices for rooting in in vitro explant culturing. Many kinds of irradiation, for example, 350–740 nm radiation [20], gamma-ray [21], and laser [22], have assisted in adventitious root induction and development in various plant species. This method for in vitro rooting without chemical utilization can effectively reduce the cost of the culture medium.

For plant tissue cultures, auxins, a group of plant growth regulators, are generally used to induce roots from the cultured explants. Without utilizing any plant hormone, Persian violet plantlets in the control group (i.e., devoid of UV-C) develop roots after 4 weeks of culture on an MS basal medium; however, the number of formed roots was only 0.2, and the length was short, only 0.04 cm long (Table 1). Thus, other means to accelerate root production in Persian violet shoots are required. In the present research, we found that a single round of low-intensity UV-C treatment for 4 h could induce root emergence of the Persian violet shoot after 2 weeks of cultivation when grown in hormone-free MS medium. Following 4 weeks of culture, the maximum root number and length were achieved from these conditions (Table 1). Therefore, a single low-intensity UV-C exposure for 4 h allowed Persian violets to develop roots before other treatments, without adding plant growth regulators to the culture medium.

On the contrary, double high-intensity UV-C exposure resulted in lower root number and length than a single exposure to low-intensity UV-C. Interestingly, when UV-C irradiation was repeated for 6 h, the root number and length were reduced by more than 5 and 17 times, respectively, compared to a single exposure (Table 1). Under these conditions, double UV-C irradiation seemed to counteract the effect of single UV-C irradiation. The utilization of UV-C to induce rooting and the counter-effect of single and double in vitro UV-C exposure has not previously been reported.

UV-C can affect plant hormone metabolism and thus indirectly influence plant growth regulation. Many ornamental plants grown in greenhouses have shown more branching and lower height after UV-C treatment at the proper dosage. An overly low level of UV-C exposure led to no effect, whereas excessive exposure damaged plants [23,24]. In pea plants, after irradiation with low and high doses of UV-C in vivo, it was found that the level of endogenous IAA increased under low UV-C treatment, while it was reduced in high dose-treated plants [25]. Hence, a single round of low-intensity UV-C exposure might suitably induce IAA synthesis within Persian violet shoots and subsequently enhance in vitro root formation. At the same time, the double high-intensity UV-C treatment possibly inhibited this synthetic pathway, resulting in slow root production. The alteration of biomolecules involved in IAA synthesis from the shoot apex of Persian violets should be further studied under low- and high-intensity UV-C exposure to confirm or refute this proposal. If such substances are higher or lower, it is possible that low- and high-intensity UV-C rays, respectively, are involved in IAA formation and thus have different root induction effects.

In addition to root initiation, a single low-intensity UV-C exposure for 4 h resulted in the highest height of Persian violet grown in vitro, while a double UV-C exposure over 4 h led to a lower height than control shoots (Table 1). This result was consistent with some ex vitro research experiments showing that plant growth decreased with increasing UV-C intensity or duration: UV-C treated lettuce at 1.6 kJ/m^2^ had lower fresh weight and expansion width than the control and the 0.8 kJ/m^2^ irradiation group [26], whereas the height and stem diameter of young tomato plants treated with 3.8 J/m^2^ for 60 min were lower than those of the control, 10 min, and 30 min groups [27].

### 3.3. In Vitro Persian Violet Flower Mutation

Apart from plant growth, UV-C exposure can also affect in vivo flowering [24,28]. For mutation induction by radiation in plant tissue cultures, many studies have been conducted to initiate morphological variation using gamma radiation [29,30,31]. The use of acute gamma rays on an in vitro culture of Persian violet changed the floral size as well as petal color and number (4 and 6 petals) [15]. Previously, UV-C was shown to be able to induce morphological variation in the Persian violet. The optimal UV-C intensity at 21.6 kJ/m^2^ results in the leaves of irradiated explants having a dark green color and being more prominent in size than those of the control after subsequent culturing on MS medium consisting of 1 mg/L BA for 30 days [32].

In this experiment, single or double UV-C exposures were used to induce morphological variation of Persian violet flowers under sterile conditions in the absence of any plant growth regulators. Single high-intensity UV-C exposure for 4, 5, or 6 h was able to induce mutations in about 20% or more of the Persian violet flowers in the total mutation count (Table S3). Moreover, when given a single high-intensity UV-C dose of up to 6 h (which was the most prolonged duration in the experiment), the maximum number of petal mutations (9) occurred, which was the highest number of petals ever reported for Persian violets. Earlier, the petal number of this plant cultured in vitro increased to 6 after acute gamma radiation exposure [15], whereas high-intensity UV-C exposure for 4 h has induced a 7-petal Persian violet [17].

It is noteworthy that 44 patterns of mutated flowers emerged after UV-C irradiation. These patterns were distinct from each other with 4 to 9 petals observed in these mutations. For the variation in petal number, mainly 6 petals were found, comprising about half of the 44 patterns, and an irregular flower form occurred for all 5-petal mutated Persian violet flowers (Figure 2, Table 2). Double low-intensity UV-C irradiation only induced two mutated flowers and gave only 6-petal flowers; while double high-intensity UV-C irradiation produced four mutated flowers with 6-petals (Table 2). Single high-intensity UV-C irradiation for 5 and 6 h also provided greater floral size and petal number than the control (Table 3). These outcomes indicate that single high-intensity UV-C irradiation for the appropriate duration is sufficient to initiate in vitro visible mutations of Persian violet.

For shoot height, the results after irradiation with UV-C and culturing on MS basal medium for 8 weeks showed that both single low- and high-intensity UV-C exposures conferred higher shoot length compared to the control (Table 3). This finding is dissimilar to that in tomato plants grown inside a greenhouse, for which the plant height was notably reduced after irradiation with 1.0 or 2.5 kJ/m^2^ UV-C [33]. Therefore, UV-C might only improve plant height in certain species.

In the present research, we assessed the in vitro mutation of Persian violet flowers by UV-C irradiation using morphological changes as morphological markers. Molecular studies, such as DNA fingerprinting, will be further conducted to demonstrate molecular variations (with respect to molecular markers) in this plant. Moreover, the stability of this flower’s morphological variation after repeated sub-culturing is an interesting point for additional investigation. This future research is expected to provide more knowledge regarding the use of UV-C to control plant growth and development under in vitro conditions.

## 4. Materials and Methods

### 4.1. Plant Materials

The in vitro clean culture of Persian violet was established by excising microshoots (5 mm long) and placing them on MS basal semi-solid medium followed by culturing for 6 weeks in a primary growth room under light (20.87 µmol/m^2^/s) and dark periods (16/8 h, respectively) at 25 ± 2 °C. After multiple shoots were obtained, the microshoots were used for the next step.

### 4.2. In Vitro Persian Violet Root Initiation

Microshoots (2–3 mm long) of Persian violet obtained from multiple shoot induction were placed on an MS basal medium and then exposed to UV-C at intensities of 30 (low-intensity) and 234 (high-intensity) µW/cm^2^ (determined by UV-C Meter, Solarmeter^®^ version 8.0, Solar Light Company Inc.) for 0, 1, 2, 4, 5, or 6 h with single or double (repeated with 24 h interval) rounds of irradiation before being transferred into the primary growth room. There were a total of three replicates for each condition (7 samples in each replicate). For double UV-C irradiation, the explants were re-irradiated 24 h after the first irradiation. The root length, number of roots, height, and number of Persian violet shoots were examined after culture for 4 weeks.

### 4.3. In Vitro Mutation of the Persian Violet Flower

After multiple shoot induction, microshoots (2–3 mm long) of Persian violet were placed on MS basal medium. Subsequently, the explants were singly or doubly (repeatedly) exposed to UV-C at 30 (low-intensity) and 234 (high-intensity) µW/cm^2^ for 0, 1, 2, 4, 5, or 6 h (for double UV-C irradiation, the explants were re-irradiated 24 h after the first irradiation) before being transferred into a primary growth room. Three replicates (with 20 samples each) were performed. After 8 weeks of cultivation, flower morphology was examined to determine the flower mutation percentage. Flowers with 5 regular petals were classified as ordinary flowers. Apart from this, a mutation was deemed to have occurred if the number of petals was higher or lower than usual, changes in petal shape had occurred, the petals had a darker or lighter color (identified and described using the RHS color chart; The Royal Horticultural Society, London, UK), the flower was larger or smaller in size, changes in the number and color of stamen were observed, and/or there were changes in the style length. The number of flowers per plantlet and the plantlet height were also investigated.

### 4.4. Statistical Analysis

Data obtained from all experiments were statistically determined using SPSS version 26 software (IBM) by calculating the mean and standard deviation and analyzing the variance (ANOVA) according to Duncan’s multiple range test with a 95% confidence level.

## 5. Conclusions

Root and flower induction from Persian violet shoots obtaining by microshoot culture as an explant were conducted under hormone-free conditions. In this research, UV-C irradiation played an innovative role in enhancing Persian violet rooting and flowering in vitro. Single or double (repeated) UV-C irradiation had different effects on the induction of Persian violet roots under sterile conditions. The highest root number and length were observed after a single low-intensity UV-C exposure for 4 h, while a lower root number and length were obtained after double high-intensity UV-C. Single or double exposure to UV-C also caused distinct morphological variations in the in vitro Persian violet flowers. Single high-intensity UV-C exposure for 5 h led to the highest percentage of floral mutation, while no mutated flower was found after a single round of low-intensity UV-C exposure. Hence, there were different responses to low- and high-intensity UV-C irradiation, in terms of variations in the in vitro root initiation and flowers of Persian violet. For this plant species, a single low-intensity UV-C exposure was found to be suitable for rooting, whereas a single high-intensity UV-C exposure was appropriate for inducing visible mutations in the flower. The application of these findings may help generate novel plants, implications in the bouquet industry, and future research.

## Figures and Tables

**Table 1:** Effect of low- and high-intensity UV-C with single or double irradiation for various durations on Persian violet shoot number, shoot height, root number, and root length after subsequent culturing for 4 weeks.

Light Intensity	No. of Irradiations	Duration (h)	Shoot Number	Shoot Height (cm)	Root Number	Root Length (cm)
Control	-	-	1.1 ± 0.2 c	1.6 ± 0.1 cd	0.2 ± 0.4 f	0.04 ± 0.1 h
Low	single	1	1.0 ± 0.0 c	1.5 ± 0.7 cde	1.2 ± 1.1 bc	1.6 ± 1.8 de
		2	1.0 ± 0.0 c	1.8 ± 0.4 c	1.4 ± 1.3 b	1.6 ± 1.8 de
		4	1.4 ± 0.9 a	3.5 ± 0.6 a	3.1 ± 1.0 a	9.3 ± 1.8 a
		5	1.1 ± 0.2 ab	3.2 ± 0.4 ab	2.7 ± 1.0 a	7.3 ± 1.3 b
		6	1.5 ± 0.7 a	3.2 ± 0.7 b	2.8 ± 0.8 a	6.3 ± 1.0 c
	double	1	1.1 ± 0.3 c	1.0 ± 0.7 gf	1.1 ± 0.8 bcd	0.7 ± 0.6 fgh
		2	1.2 ± 0.4 c	1.8 ± 0.8 c	1.5 ± 1.1 b	2.9 ± 1.7 d
		4	1.4 ± 0.6 c	1.2 ± 0.3 ef	1.2 ± 0.5 bc	1.5 ± 1.4 de
		5	1.1 ± 0.3 c	1.0 ± 0.6 gf	0.9 ± 0.8 bcd	1.3 ± 1.3 def
		6	1.0 ± 0.0 c	0.8 ± 0.5 gh	0.5 ± 0.8 de	0.4 ± 0.6 gh
High	single	1	1.0 ± 0.0 c	1.1 ± 0.3 gf	1.0 ± 1.0 bcd	1.4 ± 1.3 def
		2	1.0 ± 0.0 c	1.2 ± 0.6 gf	1.4 ± 1.0 b	1.9 ± 0.7 e
		4	1.0 ± 0.0 c	1.3 ± 0.8 def	1.2 ± 0.5 bc	2.9 ± 0.5 d
		5	1.1 ± 0.4 c	1.6 ± 0.8 cd	1.2 ± 1.0 bc	1.7 ± 1.5 e
		6	1.0 ± 0.0 c	1.3 ± 0.3 def	1.1 ± 0.8 cd	1.3 ± 0.9 def
	double	1	1.1 ± 0.3 c	1.3 ± 0.7 def	0.7 ± 0.6 cde	0.8 ± 0.8 efg
		2	1.1 ± 0.2 c	1.4 ± 0.4 def	1.1 ± 0.6 bcd	0.9 ± 0.5 efg
		4	1.0 ± 0.0 c	1.2 ± 0.3 gf	1.1 ± 0.8 bcd	0.7 ± 0.6 fgh
		5	1.0 ± 0.0 c	1.1 ± 0.5 gf	0.5 ± 1.0 de	0.4 ± 0.7 gh
		6	1.0 ± 0.0 c	0.6 ± 0.4 h	0.2 ± 0.4 f	0.04 ± 0.1 h

Values are means ± SD; means followed by the same letters within a column are not significantly different (*p* < 0.05) according to Duncan’s multiple range test (DMRT).

**Table 2:** Characteristics of 44 mutated flowers of Persian violet after irradiation with low (L)- and high (H)-intensity UV-C for single or double application for various durations.

Code	Intensity	No. of Irradiations	Duration (h)	Floral Diameter (cm)	Petal No. and Flower Form	Petal Color (Violet- Blue Group)	No. of Anthers	Anther Color (Yellow- Orange Group)	Style Length (mm)
c	-	-	-	0.8 ± 0.7	5, control	93-B	5	14-A	4
4a	H	single	6	1.4	4, standard	93-B	5	15-B	4
4b	H	single	5	1.5	4, irregular	93-C	4	14-A	4
4c	H	single	5	1.2	4, irregular	93-C	3	12-A	2
4d	H	single	5	1.4	4, irregular	93-C	5	14-A	4
5a	H	single	4	0.5	5, irregular	93-B	5	12-B	<2
5b	H	single	5	0.8	5, irregular	93-B	5	14-A	4
5c	H	single	4	1.0	5, irregular	93-B	5	14-A	<2
5d	H	single	4	1.2	5, irregular	93-C	5	12-B	4
5e	H	single	6	1.3	5, irregular	93-C	3	14-A	2
5f	H	single	6	1.3	5, irregular	93-C	5	14-A	4
5g	H	single	5	1.2	5, irregular	93-C	5	14-A	2
6a	H	single	6	1.2	6, standard	93-B	6	14-A	4
6b	H	single	6	1.4	6, standard	93-B	6	14-A	4
6c	H	single	6	1.4	6, standard	93-B	6	14-A	<2
6d	H	single	6	1.5	6, standard	93-B	7	14-A	3
6e	H	single	5	1.4	6, standard	93-B	5 (2 nm and 3 ab)	14-A	2
6f	H	single	5	1.4	6, standard	93-C	6	14-A	<2
6g	H	single	5	1.5	6, standard	93-C	6	14-A	4
6h	H	single	5	1.4	6, standard	93-B	6	14-A	<2 (2 styles)
6i	H	single	5	1.3	6, standard	93-C	6	14-A	<2
6j	H	single	5	1.3	6, standard	93-B	5	14-A	<2
6k	H	single	4	1.3	6, standard	93-C	6	14-B	4
6l	H	single	4	1.3	6, standard	93-B	6	14-B	4
6m	H	single	4	1.4	6, standard	93-C	6	15-B	4
6n	H	double	2	1.2	6, standard	93-B	5	14-A	4
6o	H	double	4	1.3	6, standard	94-C	6	14-A	2
6p	H	double	5	1.3	6, standard	94-B	6	14-B	4
6q	H	double	5	1.2	6, standard	94-C	5	14-B	4
6r	L	double	4	1.1	6, standard	94-C	5	14-B	4
6s	L	double	5	1.3	6, standard	93-B	4	14-A	2
6t	H	single	4	1.3	6, irregular	93-B	6	14-A	4
6u	H	single	5	1.0	6, irregular	94-C	5	14-A	3
6v	H	single	5	1.4	6, irregular	93-C	6	14-A	<2
7a	H	single	6	1.4	7, irregular	93-B	7	14-A	2
7b	H	single	6	1.3	7, standard	93-B	6	14-A	3
7c	H	single	5	1.5	7, standard	94- B	7	14-A	3
7d	H	single	4	1.2	7, standard	94-C	7	14-B	<2
7e	H	single	4	1.4	7, standard	94-C	7	14-B	4
7f	H	single	4	1.4	7, standard	93-B	7	14-B	4
7g	H	single	4	1.4	7, irregular	94-C	7	14-A	4
8a	H	single	5	1.3	8, irregular	93-B	8	14-A	2
8b	H	single	5	1.3	8, standard	93-B	8	14-A	4 (2 styles)
9a	H	single	6	1.4	9, irregular	93-B	3	14-A	2
9b	H	single	6	1.5	9, irregular	94-C	9	14-A	2

The code of each flower is determined according to Figure 2. For flower form, standard = similar form as control: irregular = dissimilar form to control. For the anthers, nm = normal; ab = abnormal.

**Table 3:** Effect of low- and high-intensity UV-C with single or double irradiation for various durations on Persian violet floral size, number of flowers, number of petals, and shoot height after subsequent culturing for 8 weeks.

Light Intensity	No. of Irradiations	Duration (h)	Floral Size (cm)	Number of Flowers	Number of Petals	Shoot Height (cm)
Control	-	-	0.8 ± 0.7 c	0.6 ± 0.5 c	2.9 ± 2.5 b	2.3 ± 0.2 ef
Low	single	1	0.7 ± 0.7 c	0.6 ± 0.5 c	2.8 ± 2.5 b	2.2 ± 0.1 fg
		2	0.7 ± 0.7 c	0.6 ± 0.5 c	2.9 ± 2.5 b	2.3 ± 0.2 ef
		4	0.8 ± 0.7 c	0.6 ± 0.5 c	3.0 ± 2.5 b	4.1 ± 0.3 a
		5	0.8 ± 0.7 c	0.6 ± 0.5 c	2.9 ± 2.5 b	4.0 ± 0.3 a
		6	0.7 ± 0.7 c	0.6 ± 0.5 c	2.8 ± 2.5 b	3.8 ± 0.2 b
	double	1	0.9 ± 1.7 c	0.5 ± 0.5 c	2.7 ± 2.5 b	2.1 ± 0.1 h
		2	0.8 ± 0.7 c	0.6 ± 0.5 c	2.9 ± 2.5 b	2.4 ± 0.2 e
		4	0.9 ± 0.7 bc	0.6 ± 0.5 c	3.2 ± 2.4 b	2.3 ± 0.2 ef
		5	1.0 ± 1.8 bc	0.6 ± 0.5 c	3.2 ± 2.5 b	2.2 ± 0.3 fg
		6	0.9 ± 0.6 bc	0.7 ± 0.5 bc	3.4 ± 2.4 b	2.2 ± 0.3 gh
High	single	1	0.8 ± 0.7 c	0.6 ± 0.5 c	3.0 ± 2.5 b	2.2 ± 0.2 fg
		2	0.9 ± 0.6 bc	0.7 ± 0.5 bc	3.3 ± 2.4 b	2.3 ± 0.2 efg
		4	1.1 ± 0.5 bc	0.9 ± 0.3 b	4.5 ± 1.9 a	3.8 ± 0.4 b
		5	1.3 ± 0.3 ab	1.0 ± 0.2 a	5.1 ± 1.2 a	3.8 ± 0.6 b
		6	1.6 ± 2.3 a	0.9 ± 0.3 b	4.9 ± 1.6 a	3.7 ± 0.5 b
	double	1	0.9 ± 0.6 bc	0.7 ± 0.5 bc	3.6 ± 2.3 b	2.4 ± 0.3 ef
		2	0.9 ± 0.6 bc	0.7 ± 0.5 bc	3.4 ± 2.4 b	3.4 ± 0.7 d
		4	1.1 ± 1.7 bc	0.7 ± 0.5 bc	3.5 ± 2.3 b	3.6 ± 0.3 c
		5	0.9 ± 0.6 bc	0.7 ± 0.5 bc	3.6 ± 2.3 b	3.7 ± 0.2 bc
		6	0.9 ± 0.6 bc	0.7 ± 0.5 bc	3.6 ± 2.3 b	2.3 ± 0.4 ef

Values are means ± SD; means followed by the same letters within a column are not significantly different (*p* < 0.05) according to Duncan’s multiple range test (DMRT).

## Data Availability

Data is contained within the article and supplementary materials.

## Supplementary Materials

The following are available online at https://www.mdpi.com/article/10.3390/plants10122671/s1, Table S1: Effect of low- and high-intensity UV-C with single or double irradiation for various durations on the number of ordinary and mutated Persian violet flowers after subsequent culturing for 8 weeks.; Table S2: Effect of low- and high-intensity UV-C with single or double irradiation for various durations on Persian violet flower percentage after subsequent culturing for 8 weeks.; Table S3: Effect of low- and high-intensity UV-C with single or double irradiation at various durations on the mutation percentage of Persian violet flowers after subsequent culturing for 8 weeks.
