# Supplementary material for: Differing In Vitro Rooting and Flowering Responses of the Persian Violet to Low and High UV-C Irradiation
Source: Plants (Basel). 2021 Dec 4;10(12):2671. doi: 10.3390/plants10122671 (PMC8706140; doi:10.3390/plants10122671)
Supplement: Supplementary file 1 [file plants-10-02671-s001.zip › plants-1448159-supplementary.pdf]

plants

MDPI

Table S1. Effect of low- and high-intensity UV-C with single or double irradiation for various durations on the number of ordinary and mutated Persian violet flowers after subsequent culturing for 8 weeks.

Intensity	No. of Irradiations	Type of Flower	Time of Irradiation (h)					
			0	1	2	4	5	6
Low	single	Ordinary	35	33	35	36	35	34
		Mutated	-	-	-	-	-	-
		Total	35	33	35	36	35	34
	double	Ordinary	35	32	35	38	37	39
		Mutated	-	-	-	-	1	1
		Total	35	32	35	38	38	40
High	single	Ordinary	35	35	39	41	42	45
		Mutated	-	-	-	11	16	11
		Total	35	35	39	52	58	56
	double	Ordinary	35	43	40	41	42	42
		Mutated	-	-	1	1	1	1
		Total	35	43	41	42	43	43

Table S2. Effect of low- and high-intensity UV-C with single or double irradiation for various durations on Persian violet flower percentage after subsequent culturing for 8 weeks.

Intensity	No. of Irradiations	Time of Irradiation (h)					
		0	1	2	4	5	6
Low	single	58.3	55	58.3	60	58.3	56.7
	double	58.3	53.3	58.3	63.3	63.3	66.7
High	single	58.3	58.3	65	68.3	86.7	93.3
	double	58.3	71.7	68.3	70	71.7	71.7

The formula for calculating flower percentage is as follows: Flower percentage = (A/60) × 100%, where A is the total number of flowers produced in treatment.

Table S3. Effect of low- and high-intensity UV-C with single or double irradiation at various durations on the mutation percentage of Persian violet flowers after subsequent culturing for 8 weeks.

**Intensity**	**No. of Irradiations**	**Time of Irradiation (h)**					
		0	1	2	4	5	6
Low	single	0	0	0	0	0	0
	double	0	0	0	0	2.6	2.5
High	single	0	0	0	21.2	27.6	19.6
	double	0	0	2.4	2.4	2.3	2.3

The formula for calculating mutated flower percentage is as follows: Mutated flower percentage = (A/B) × 100%, where A is the total number of mutated flowers produced in treatment, and B is the total number of flowers produced in treatment.
